# Assessment of the Prevalence and Level of Awareness of Medication Overuse Headache Among the General Population in Makkah City, Saudi Arabia

**DOI:** 10.7759/cureus.37985

**Published:** 2023-04-22

**Authors:** Abdullah S Alharbi, Omar F Alharbi, Fadi L Qutub, Warif M Albogami, Mohammed A Aljuhnie, Abdullah E Alharbi, Wed N Alqahtani, Omar Babateen

**Affiliations:** 1 Department of Medicine and Surgery, College of Medicine, Umm Al-Qura University, Makkah, SAU; 2 Department of Physiology, College of Medicine, Umm Al-Qura University, Makkah, SAU

**Keywords:** saudi arabia, general population, knowledge, chronic headache, prevalence, medication overuse headache, awareness, makkah

## Abstract

Background: Medication overuse headache (MOH) is a secondary headache condition caused by consistently using more medication than necessary to treat headache symptoms. MOH is defined as a headache that occurs for 15 or more days per month in a patient with a pre-existing primary headache, and it develops as a result of regular overuse of symptomatic headache medication for more than three months. Patients with headaches often use simple pain medication for 15 or more days per month (e.g., non-steroidal anti-inflammatory drugs (NSAIDs) and paracetamol) and 10 or more days per month of opioids, triptans, and combination analgesics, but when there is no relief from these medications, the headache progression can lead to a cycle of consuming more medication with increased pain, which can lead to MOH.

Objective: This study aimed to assess the prevalence and awareness of MOH among the general population of Makkah, Saudi Arabia.

Methods: A cross-sectional study was conducted between December 2022 and March 2023 using a self-administered online questionnaire disseminated through social media. Data were collected from females and males 18 years of age and older living in Makkah, Saudi Arabia.

Results: Overall, 715 individuals completed the questionnaire, 497 of whom were female (69.5%). The average age of the participants was 32.9 years (±13.3 years). The prevalence of MOH among those who reported having experienced headaches throughout their lifetimes was estimated to be 4.5%. Only 134 people (18.7%) were determined to be aware of MOH.

Conclusion: This study demonstrated that the general population of Makkah has a high prevalence of MOH and low levels of MOH awareness.

## Introduction

Headaches are a prevalent and significant public health issue [1]. According to the third edition of the International Classification of Headache Disorders (ICHD-3), migraines and tension-type headaches (TTH) are the two most common forms of primary headaches [1]. Patients with headaches often use simple pain medication for 15 or more days per month (e.g., non-steroidal anti-inflammatory drugs (NSAIDs) and paracetamol) and 10 or more days per month of opioids, triptans, and combination analgesics, but when there is no relief from these medications, the headache progression can lead to a cycle of consuming more medication with increased pain, which can lead to medication overuse headaches (MOH), also known as medicine-misuse headaches [2]. These occur when the medication used to treat a headache becomes the cause of the headache [2]. MOH is classified as a secondary headache disease according to ICHD-3 and was ranked 20th in 2015 for the Global Burden of Disease (GBD) [3]. It is caused by more frequent use of medication than necessary to treat headache symptoms [4]. It was not identified as an independent condition in the most recent GBD studies released in 2019 but rather as a contributor to the burden of migraine and TTH [1].

MOH is defined as a headache that occurs for 15 or more days per month in a patient with a pre-existing primary headache, and it develops as a result of regular overuse of symptomatic headache medication for more than three months [5]. MOH can be defined differently depending on the medication and is measured in terms of both duration of use and number of treatment days per month [6]. Studies show that MOH has an association with the use of analgesics, including paracetamol, NSAIDs, opioids, triptans, and combination analgesics, for 10 or more days per month [3]. There is now strong evidence that all headache medications can lead to the development of MOH in people with primary headache illnesses [7]. MOH can be a distinct form of headache or an aggravation of an old one [3]. When MOH develops in a patient who already has a headache, the patient is diagnosed with both disorders [3].

MOH has multiple known risk factors, including sedative usage, musculoskeletal or gastrointestinal issues, anxiety, depression, a sedentary lifestyle, smoking, and migraines [5]. The risk factors for MOH are mostly determined by the kind of medication being misused, with opioids, barbiturates, or combination analgesics being more likely to result in MOH than triptan or ergotamine usage [5]. The type of prior headache also affects the chance of MOH developing [5]. The pre-existing headache in the majority of instances is a migraine, while MOH is less common in individuals with TTH or cluster headaches [5]. Asthenia, gastrointestinal problems, irritability, anxiety, restless limbs, sadness, memory changes, and difficulties concentrating are all possible symptoms associated with MOH [4]. The headaches are persistent, happen regularly, are severe, and may shift location on various days [6].

MOH is found in 1%-2% of the general population globally [6]. It affects over 63 million people worldwide [4]. MOH is present in around 50% of chronic headache cases, with the highest frequency in middle-aged patients, particularly those in the fourth and fifth decades of life [3,5]. According to studies, women are more susceptible to MOH than males, with a 3.5:1 ratio, which is greater than expected based on sex differences [4]. A previous study on MOH in the Qassim Province of Saudi Arabia found a prevalence of MOH of 4% and that MOH awareness was 18% [4]. A study conducted in the UK found that 77% of people were unaware of the possibility of developing MOH from regular analgesic use for headaches [6].

There have been no studies on the prevalence and awareness of MOH in Makkah. Our study aims to fill this gap by assessing MOH prevalence and awareness among the general population of Makkah, Saudi Arabia.

## Materials and methods

Study design and participants

This observational cross-sectional study aimed to investigate the prevalence and awareness level of MOH among the general population in Makkah, Saudi Arabia. To recruit participants, convenience sampling was used. Females and males 18 years of age and older who were living in Makkah were included. The study excluded participants who declined to participate or those who were under the age of 18.

Ethical considerations and sample size

This study was conducted between December 2022 and March 2023 in Makkah, Saudi Arabia. After ethical approval was obtained from Umm Al-Qura University’s Biomedical Ethics Committee (Approval No. HAPO-02-K-012-2023-01-1379), a self-administered online questionnaire was disseminated via social media channels to the general population. The minimum required sample size in this study was 385 participants, keeping a 95% confidence interval and a p-value of 5%, as calculated by the OpenEpi (version 3.01) website [8]. The total number of participants recruited in this study was 715 to optimize the results' generalizability and accuracy.

Study tool and scoring system

The survey was developed using the ICHD-3 diagnostic criteria for MOH, which were also applied in previous studies done in Qassim and Riyadh [4,9]. A pilot study was also conducted to ensure simplicity and clarity. The questionnaire form consisted of 10 multiple-choice questions and was divided into three sections. The first section gathered participants’ demographic data (age, gender, educational level, and nationality). The second section assessed the prevalence of headaches among the population studied by asking if they had ever had a headache before and how many days they had had headaches in the previous 30 days. The third section asked about the type of medications used to relieve their headaches, the number of days they used their headache medication within the last 30 days, and, to determine participant awareness of MOH, what the headache medication’s side effects were.

The diagnosis of MOH in this study was determined by using a scoring system based on the participants’ responses to four questions. The total score for these questions was 11, and participants were presumed to have MOH if their responses added up to a minimum of eight points (Table 1).

**Table 1:** Scoring system for diagnosing MOH MOH: medication overuse headache

Question	No points	One point	Two points	Three points	Four points
For how many days have you experienced headaches within the last 30 days?	Did not experience any headaches	1-2 days	3-7 days	8-14 days	15 days or more
Which of the following medications do you use to relieve your headache?	Does not use medications	Use medications			
For how many days have you used these medications to relieve your headache within the last 30 days?	Did not use headache medications within the last 30 days	1-9 days	10-14 days	15-30 days	
For how long do you usually use these medications to relieve your headache?	Intermittently	Less than a month	1-3 months	More than 3 months	

The first question in the scoring system was, “For how many days have you experienced headaches within the last 30 days?” The response “I did not experience any headaches within the last 30 days” was scored as zero, “one to two days” as one, “three to seven days” as two, “eight to 14 days” as three, and “15 days or more” as four. The second question was, “Which of the following medications do you use to relieve your headache?” Taking paracetamol, NSAIDs, Solpadeine, and/or sumatriptan for headaches was scored as one. The third question was, “For how many days have you used these medications to relieve your headache within the last 30 days?” The response “Did not use headache medications within the last 30 days” was scored as zero, “one to nine days” as one, “10 to 14 days” as two, and “15 to 30 days” as three. The last question was, “For how long do you usually use these medications to relieve your headache?” The answer “intermittently” was scored as 0, “less than one month” as one, “one to three months” as two, and “more than three months” as three. Anyone who scored eight or more points overall was presumed to have MOH. The survey also assessed MOH awareness by including a question with multiple choices regarding the side effects of the previously stated medications, with chronic headaches as one of the side effects. Participants were assumed to be aware of MOH if they selected chronic headaches as a side effect.

Statistical analysis

The obtained data were analyzed using the IBM Corp. Released 2019. IBM SPSS Statistics for Windows, Version 26.0. Armonk, NY: IBM Corp. The mean and standard deviation were used to represent quantitative data, and qualitative data were represented as frequencies and percentages.

## Results

Overall, 715 individuals completed the questionnaire, 497 of whom were female (69.5%). The vast majority were Saudi nationals (95.1%). A bachelor's degree was the most common highest level of education among the participants (57.6%), followed by a high school diploma (30.6%). Participants' average age was 32.9 years (±13.3 years). Additional demographic data are represented in Table 2.

**Table 2:** Demographic characteristics of the participants

Variable	Choices	Frequency	Percentage
Gender	Female	497	69.5%
	Male	218	30.5%
	Total	715	100%
Nationality	Saudi	680	95.1%
	non-Saudi	35	4.9%
	Total	715	100%
Educational level	Bachelor's degree	412	57.6%
	High school	219	30.6%
	Less than high school	29	4.1%
	Master's degree	44	6.2%
	PhD degree	11	1.5%
	Total	715	100%
Age	Mean	32.94	
	Standard deviation	13.3	

Out of the 715 participants, 669 (93.6%) reported having headaches at some point in their lives. Reporting on the 30 days before taking the survey, 289 people (43.2%) had headaches lasting only one or two days, 180 people (26.9%) had headaches lasting three to seven days, 54 people (8.1%) had headaches lasting eight to 14 days, and 38 people (5.7%) had headaches lasting more than 15 days. Among those surveyed, 108 (16.1%) reported being headache-free within the past 30 days (Table 3).

**Table 3:** Participants’ headache history

Question	Choices	Frequency	Percentage
Have you ever had a headache in the past?	yes	669	93.6%
	no	46	6.4%
	Total	715	100%
For how many days have you experienced headaches within the last 30 days?	1 - 2 days	289	43.2%
	3 - 7 days	180	26.9%
	8 - 14 days	54	8.1%
	15 days or more	38	5.7%
	I did not experience any headaches within the last 30 days	108	16.1%
	Total	669	100%

Among those who had experienced headaches, 130 individuals (19.4%) did not use any medicine for headache relief. Among those who used medication, the majority of participants (74.9%) preferred paracetamol for headache relief. Solpadeine was the second most common medicine among participants (18.5%), followed by NSAIDs (11.7%). Sumatriptan was the least common medication among participants (Table 4).

**Table 4:** Types of headache medication(s) used by the participants NSAIDs: non-steroidal anti-inflammatory drugs

Question	Medications (participants can choose more than one)	Frequency	Percentage
Which of the following medications do you use to relieve your headache?	I don’t use medications for headache	130	19.4%
	Paracetamol (Panadol, Fevadol, Adol, Panadrex, or Acitam)	501	74.9%
	NSAIDs (aspirin, ibuprofen, or diclofenac)	78	11.7%
	Solpadeine	124	18.5%
	Sumatriptan	12	1.8%

In the last 30 days, 40.2% of people who had headaches did not take any medication to relieve their headaches. Among the 59.8% who took medications in the last month, 49.9% used them for one to nine days, 5.1% for 10 to 14 days, and 4.8% for more than 15 days. In terms of medication usage frequency, the majority of respondents (75.3%) used medications on an as-needed basis, 9% used them for less than a month, 6.9% used them for one to three months, and 9.3% used them for more than three months (Table 5).

**Table 5:** Participants' history of headache medication(s)

Question	Choices	Frequency	Percentage
For how many days have you used these medications to relieve your headache within the last 30 days?	Did not use headache medications within the last 30 days	269	40.2%
	1-9 days	334	49.9%
	10-14 days	34	5.1%
	15-30 days	32	4.8%
	Total	669	100%
For how long do you usually use these medications to relieve your headache?	Intermittently	504	75.3%
	Less than one month	60	9%
	1-3 months	43	6.4%
	More than three months	62	9.3%
	Total	669	100%

Table 6 demonstrates the results of the scoring system. Applying this system, the prevalence of MOH among those who reported having experienced headaches throughout their lifetimes was estimated to be 4.5%.

**Table 6:** The results of the scoring system

Score	Frequency	Percentage
0	20	3%
1	80	12%
2	94	14.1%
3	174	26%
4	152	22.7%
5	70	10.5%
6	28	4.2%
7	21	3.1%
8	15	2.2%
9	4	0.6%
10	8	1.2%
11	3	0.4%
Total	669	100%

In terms of MOH awareness, only 134 people (18.7%) were considered aware of MOH based on their identification of chronic headaches as a medication side effect (Table 7).

**Table 7:** Participants awareness of the side effects caused by headache medications

Side effects (participants can choose more than one)	Frequency	Percentage
Diarrhea	56	7.8%
Increased sweating	136	19%
Loss of appetite	169	23.6%
Skin rash	36	5%
Tiredness or weakness	303	42.4%
Fever	86	12%
Bloody urine	33	4.6%
Chronic headache	134	18.7%
Stomach cramps	225	31.5%

## Discussion

MOH is one of the most prevalent chronic headache conditions, affecting 1% to 2% of the world's population [3]. As such, we aimed to determine the prevalence and awareness level of MOH among the general population in Makkah, Saudi Arabia, in this study.

This study found a prevalence of MOH of 4.5%. Another study done in Iran reported a prevalence of MOH of 4.9%, which is somewhat in agreement with this study’s findings [10]. Our result is inconsistent with previous studies. For example, a higher prevalence of MOH was reported in Zambia (7.1%) and Russia (7.2%), while a lower prevalence of MOH was reported in previous studies done in Norway (1.7%), Spain (1.4%), Ethiopia (0.7%), and Lithuania (3.2%) [11-16].

The discrepancy between the findings of this study and the study done in Zambia could be explained by the fact that in Zambia, there is a culture of turning to over-the-counter analgesics that is unrestrained by any public health education due to the lack of access to healthcare and the poor knowledge of managing headache disorders among the few healthcare workers available [11]. For the study done in Norway, a physical examination and an interview were performed by two neurological residents trained and experienced in headache diagnostics, while in the study from Spain, complete physical, neurological, and ophthalmological examinations, as well as blood tests and neuroimaging, were performed. For the studies done in Ethiopia, Lithuania, and Russia, the different sampling methodologies used could explain the inconsistency with our findings. The various sampling criteria, diagnostic parameters, and methodologies used in the studies may be the cause of the discrepancy between our findings and those of others regarding the prevalence of MOH [17].

The level of awareness of MOH reported in the current study was 18.7%. Another study done in Saudi Arabia reported MOH awareness at 18%, which is somewhat in agreement with this study’s findings [4].

In the current research, paracetamol was the most frequently used analgesic (74.9%); previous studies done in Saudi Arabia, Denmark, and Sweden reported similar findings [4,18,19]. This is unsurprising because paracetamol has broad therapeutic uses, is well tolerated, and is available in different pharmaceutical forms [20].

In the present research, MOH was more prevalent in women, which is probably because migraine, a predisposing factor for MOH, is more prevalent in women [21,22].

This is the first study conducted in Makkah addressing the prevalence and awareness of MOH and is one of the few studies done in Saudi Arabia, with a larger sample size of 715 participants. The goal was to obtain results that were precise, reliable, and satisfying. Nevertheless, during this study, some limitations were noted. One limitation was that, for data collection, the study used a self-reported questionnaire. This raises the probability of recall bias. Another limitation was that females (69.5%) made up the majority of the study's participants, which has an impact on how accurately the results are generalized. Also, the majority of participants had a bachelor’s degree or higher. As such, it was difficult to assume that the results were representative of the entire population, as it is likely that the sampling of relatively well-educated respondents resulted in an overestimation of population-level awareness. Because of this, awareness may be lower than this study found. Therefore, additional research on this subject involving various sociodemographic data as well as other study designs is required.

## Conclusions

This study has shown that the general population of Makkah has a high prevalence of MOH and a low level of awareness. Due to the high prevalence of MOH, the study suggests performing community-based local health awareness programs, campaigns, and workshops to increase public awareness. Additionally, the study highlights the importance of limiting the overuse of headache medications to prevent MOH and of seeking medical consultation to develop a thorough treatment plan if headaches do occur.
